# A novel c.5308_5311delGAGA mutation in Senataxin in a Cypriot family with an autosomal recessive cerebellar ataxia

**DOI:** 10.1186/1471-2350-9-28

**Published:** 2008-04-14

**Authors:** Paschalis Nicolaou, Anthi Georghiou, Christina Votsi, Lefkos T Middleton, Eleni Zamba-Papanicolaou, Kyproula Christodoulou

**Affiliations:** 1 The Cyprus Institute of Neurology and Genetics, 6 International Airport Avenue, Nicosia, Cyprus; 2 Imperial College, London, UK

## Abstract

**Background:**

Senataxin (chromosome 9q34) was recently identified as the causative gene for an autosomal recessive form of Ataxia (ARCA), termed as Ataxia with Oculomotor Apraxia, type 2 (AOA2) and characterized by generalized incoordination, cerebellar atrophy, peripheral neuropathy, "oculomotor apraxia" and increased alpha-fetoprotein (AFP). Here, we report a novel Senataxin mutation in a Cypriot ARCA family.

**Methods:**

We studied several Cypriot autosomal recessive cerebellar ataxia (ARCA) families for linkage to known ARCA gene loci. We linked one family (909) to the SETX locus on chromosome 9q34 and screened the proband for mutations by direct sequencing.

**Results:**

Sequence analysis revealed a novel c.5308_5311delGAGA mutation in exon 11 of the SETX gene. The mutation has not been detected in 204 control chromosomes from the Cypriot population, the remaining Cypriot ARCA families and 37 Cypriot sporadic cerebellar ataxia patients.

**Conclusion:**

We identified a novel SETX homozygous c.5308_5311delGAGA mutation that co-segregates with ARCA with cerebellar atrophy and raised AFP.

## Background

Autosomal recessive cerebellar ataxias (ARCA) form a group of heterogeneous disorders characterised by autosomal recessive inheritance, cerebellar ataxia and an early onset of disease, usually before the age of 20 [1]. The so called Ataxia with Oculomotor Apraxia type 2 was recently found as the second most frequent ARCA, after Freidreich's Ataxia in a group of 77 families, most of them (69%) of French origin [2]. AOA2 has been mapped to chromosome 9q34 [3,4] and more recently mutations in the senataxin (SETX) gene were identified in AOA2 patients [5]. Senataxin contains a domain found in superfamily 1 of helicases and is the human ortholog of yeast Sen1p gene [5]. Autosomal dominant mutations in the same gene occur in a form of juvenile amyotrophic lateral sclerosis (ALS4) [6].

In the late 1980s a cluster of 7 Friedreich's Ataxia families with 13 affected members was identified in the Cypriot population and the highest estimated carrier frequency of 1-in-6 to 1-in-7 was reported for two neighbouring villages in the western part of the island [7]. Since then 16 additional FRDA patients, 13 non-FRDA ARCA families, 1 ADSCA family and 37 sporadic cerebellar ataxia patients were ascertained in the Cypriot population.

Initial linkage studies of the non-FRDA ARCA families showed linkage of family 909 to the senataxin locus on chromosome 9q34. We hereby report the identification of the second ARCA mutation in the Cypriot population, a novel SETX deletion. This mutation has not been identified in 204 normal chromosomes and it has not been detected in any of the remaining non-FRDA ARCA families or sporadic cerebellar ataxia patients in our population.

## Methods

### Subjects and samples

**Figure 1:**
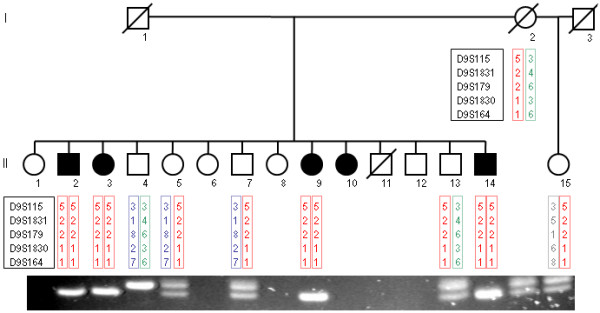
**Pedigree of the Cypriot ARCA family 909**. Haplotypes spanning the SETX locus are shown under the corresponding available family members (I-2, II-2, II-3, II-4, II-5, II-7, II-9, II-13, II-14 and II-15). Haplotypes were constructed using the order in which the microsatellite marker loci are located in the region; D9S115, D9S1831, D9S179, D9S1830 and D9S164. Affected individuals (II-2, II-3, II-9 and II-14) are homozygous for the 5-2-2-1-1 haplotype. PCR and agarose gel electrophoresis based mutation detection results are also shown below the corresponding individual. A 105 bp band denotes a normal sequence and a 101 bp band denotes a deleted sequence (c.5308_5311delGAGA). Homozygous normal individuals (II-4) have a single 105 bp band, homozygous mutant individuals (II-2, II-3, II-9 and II-14) have a single band of 101 bp and heterozygous mutation carrier individuals (I-2, II-5, II-13 and II-15) have two bands of 105 and 101 bp.

**Figure 2:**
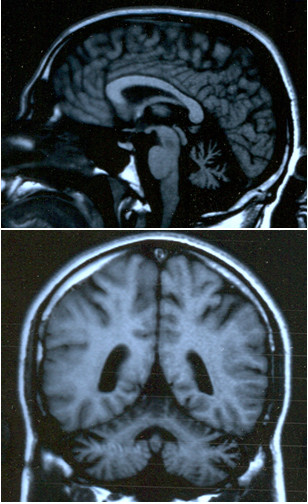
Brain MRI images of the younger patient (II-14) at age 25 years old, showing cerebellar atrophy

Family 909 was a two-generation, 18-member Greek Cypriot ataxia kindred with 5 affected individuals in one generation (Figure 1). The parents originate from different villages 10 Km apart in the Paphos district of Cyprus, and any traceable relation was excluded. Our study of ataxias in the Paphos district of Cyprus was approved by the Cyprus Institute of Neurology and Genetics Ethics Committee. Written informed consent was obtained from all participants of the study. Ten members (4 affected) of the family were examined clinically and brain MRI was performed in the youngest affected individual (II-14) at age 25 years old. The proband (II-2) at the last examination was a 44-years-old male with a 30 year history of ataxia (onset at 14 yo). He presented with ataxia, of limb and trunk movements, truncal titubation, dysarthria, loss of proprioception and vibration sense, absent deep tendon reflexes in the lower limbs, bilateral Babinski sign with pes cavus, spasticity, and scoliosis. There were spontaneous random saccadic eye movements at rest. Saccadic eye movements were present, slow but dysmetric and irregular to visual targets in all directions (horizontal and vertical). The patient had diabetes type 2 but no cardiomyopathy. Overall the clinical presentation of the affected family members included an age at onset of 8 to 14 years old, slow progressive ataxia of limb and trunk and eye movements with multidirectional nystagmus, peripheral neuropathy (clinically), spasticity, Babinski sign and areflexia. The clinical features of the four participating patients are included in Table 1. Patient II-3 had a slightly milder form of the disease with age of onset at the age of 14, less spasticity in the lower limbs and no pes cavus deformity. This patient had mild right eye esophoria. Overall, there was no significant variability of the clinical phenotype in this family. Brain MRI of the younger patient (II-14) showed cerebellar atrophy (Figure 2). Vitamin E levels were within the normal range but there was marked increase of AFP levels (Table 1). All patients refused to undergo electrophysiological studies.

**Table 1:** Clinical features of family 909 patients. The clinical characteristics of participating patients are presented below.

***Clinical feature***	***Patient***			
	***II-2***	***II-3***	***II-9***	***II-14***

Gender	M	F	F	M
Age of onset	14	14	8	8
Ataxia	+	+	+	+
Dysarthria	+	+	+	+
Abnormal eye movements	+	+	+	+
Deep tendon reflexes	areflexic	areflexic	Areflexic	areflexic
Spasticity in the lower limbs	+	+	+	+
Babinski sign	+	indifferent	+	+
Pes cavus	+	-	+	+
Deep sensation (proprioception and vibration)	reduced	reduced	Reduced	reduced
Scoliosis	+	+	+	+
Muscle weakness in upper limbs	distal	distal	Distal	distal
Muscle weakness in lower limbs	proximal + distal	proximal + distal	proximal + distal	proximal + distal
Muscle atrophy in upper limbs	distal	distal	distal	distal
Muscle atrophy in lower limbs	distal	distal	distal	distal
Cardiomyopathy	-	-	-	-
Diabetes mellitus	+	-	-	-
AFP levels in IU/ml	30.5	29.6	34.0	n/a
MRI signs of cerebellar atrophy	n/a	n/a	n/a	+

+ = presence, - = absence, n/a = not available, AFP normal range = < 5.5 IU/ml

### Molecular genetic analyses

Blood samples were obtained after informed consent from individuals I-2, II-2, II-3, II-4, II-5, II-7, II-9, II-13, II-14 and II-15. DNA was extracted using standard salting out procedures. Frataxin (FXN) gene GAA repeat expansion analysis revealed normal range bands in the proband, thus excluding the diagnosis of FRDA [8]. All available individuals were genotyped at microsatellite polymorphic marker loci, spanning candidate ARCA loci (AVED/TTPA: D8S1696, D8S544; ARSACS/SACS: D13S232, D13S292; AOA1/APTX: D9S1118 and AOA2/SETX: D9S115, D9S1831, D9S179, D9S1830, D9S164), following previously described methodology [9]. Haplotypes of individuals were constructed manually.

### Linkage analyses

Linkage analysis was performed using the LINKAGE package of programs [10]. Lod scores (*Z*) were calculated under an autosomal recessive inheritance model and a disease allele frequency of 1 in 10,000.

### Sequence analysis

Genomic DNA sequencing of the SETX gene was performed on the proband DNA. Primers amplifying the sequence of the 26 SETX exons were designed by us and are available upon request. Amplification products were sequenced in both directions using the CEQ Dye Terminator Cycle Seq. Sequence traces were automatically compared with the normal SETX gene sequence as listed in the GenBank database (Accession: NT_035014, Region: 1915823..2007712, Version: NT_035014.4, GI: 51467290 of 02 Mar 2006), using the CEQ8000 investigator software.

### Family mutation detection

A 105 bp fragment encompassing the family deletion mutation sequence was amplified using flanking primers 5'-GGCACAAGAATGGCTCAACT-3' and 5'-AACTTACCTGCAAACTCCCAGT-3'. PCR products were run on a 7% agarose gel. All available family members were initially screened for the mutation. The proband of each Cypriot non-FRDA ARCA families and all Cypriot ARCA sporadic patients were screened for the mutation at a later stage.

## Results

Initial linkage analysis of the family to the *TTPA*, *SACS*, *APTX *and *SETX *loci revealed probable linkage of the family to the *SETX *locus on chromosome 9q34 with a maximum lod score of 2.31 at recombination fraction 0 with microsatellite marker loci D9S115, D9S1831, D9S179 and D9S164 (Table 2). Haplotype analysis further supported the linkage data (Figure 1) with affected individuals sharing the same haplotype (5-2-2-1-1 for D9S115-D9S1831-D9S179-D9S1830-D9S164, respectively) in a homozygous state.

**Table 2:** Linkage analysis data of family 909 to ARCA loci. Two-point lod score values obtained between the disease in family 909 and each marker locus are presented.

***Locus***		***LOD score values at θ***				
***Gene***	***Marker***	***0.0***	***0.01***	***0.05***	***0.1***	***0.2***
***TTPA***	D8S1996	-inf.	-1.26	-0.58	-0.31	-0.09
	D8S544	-inf.	-1.74	-0.51	-0.11	0.12
***SACS***	D13S232	-inf.	-2.81	-1.45	-0.89	-0.39
	D13S292	-inf.	-2.81	-1.45	-0.89	-0.39
***APTX***	D9S1118	-inf.	-5.12	-2.46	-1.42	-0.56
***SETX***	D9S115	2.31	2.26	2.06	1.81	1.29
	D9S1831	2.31	2.26	2.06	1.81	1.29
	D9S179	2.31	2.26	2.06	1.81	1.29
	D9S1830	2.18	2.14	1.96	1.73	1.24
	D9S164	2.31	2.26	2.06	1.81	1.29

-inf. = - infinity

**Figure 3:**
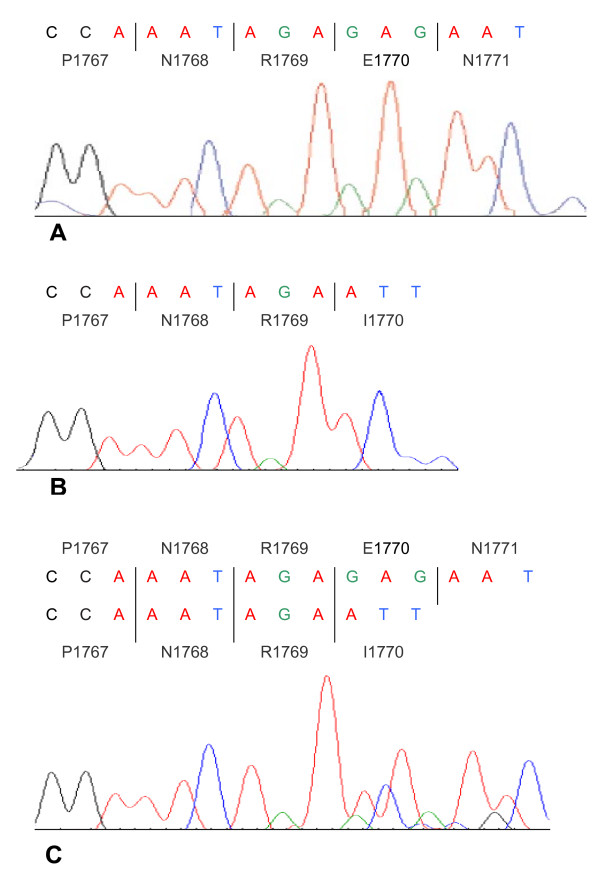
**Sequence analysis data of the SETX gene region**. **A**: amino acid sequence of normal control individual (II-4). **B**: amino acid sequence of the proband (II-2), homozygous for the c.5308_5311delGAGA mutation. **C**: amino acid sequence of carrier (I-2), heterozygous for the c.5308_5311delGAGA mutation.

We then performed sequence analysis of the proband (II-2) DNA sample for the 26 SETX exons and detected a novel homozygous SETX gene deletion c.5308-5311delGAGA (Figure 3). This 4 bp deletion causes a frame shift mutation that results in a Glu-to-Ile change at amino acid 1770 of the SETX gene and also introduces a stop codon 15 amino acids further down the new reading frame (p.Glu1770IlefsX15). We designed a PCR and agarose gel electrophoresis based assay of this mutation in order to further investigate the remaining affected and non-affected members of the family and other additional Cypriot samples. The c.5308-5311delGAGA mutation co-segregates with the disease haplotype (5-2-2-1-1) of the family and is found in a homozygous state in all four affected individuals (Figure 1). It was not detected in any of 204 normal Cypriot population chromosomes analysed, thus further supporting the hypothesis that this is the disease causing mutation in this Cypriot ARCA family.

The above novel SETX mutation is the second ARCA mutation, after the FRDA GAA repeat expansion, thus far identified in the Cypriot population. Therefore, we used the PCR based assay to investigate whether any of the remaining molecularly undiagnosed Cypriot cerebellar ataxia patients have this newly identified Cypriot ARCA mutation. The proband of 12 ARCA families and 37 sporadic SCA patients, previously excluded for the FRDA, SCA1, SCA2, SCA3, SCA6, SCA7, SCA8, SCA10, SCA12, SCA17 and DRPLA mutations, were analysed for this novel SETX mutation. The mutation was not detected in any of the above patients, thus indicating that this is a rare ARCA mutation in our population.

## Discussion

We report a novel SETX gene mutation in a Cypriot ARCA family. The clinical phenotype of the reported patients is similar to other reported SETX mutation patients. Overall the clinical presentation of the affected family members included an age at onset of 8 to 14 years old, slow progressive ataxia of limb and trunk and eye movements with multidirectional nystagmus, peripheral neuropathy (clinically), spasticity, Babinski sign and areflexia. No significant intrafamilial clinical variability was observed although one patient (II-3) had a slightly milder form of the disease. AFP levels were measured in 3 of the 5 patients (Table 1) and all were highly elevated, as characteristically found in AOA2 patients.

Senataxin encodes a ubiquitously expressed protein of 2,677 amino acids that shares similarities with the yeast Sen1 proteins mainly at the carboxy-terminus conserved superfamily I DNA/RNA helicase domain spanning amino acids 1,931 to 2,456 [5,6]. Although the function of senataxin is poorly understood, it is speculated that, like the DNA/RNA helicases, it is involved in DNA repair, replication, recombination, transcription, RNA processing, transcript stability and translation initiation. Evidence that SETX plays a role in the defence against oxidative DNA damage has recently been reported [11]. In human cultured cells SETX was found as expected in the nucleus, but also diffused in the cytoplasm, thus indicating additional functions of the protein beyond those of the yeast Sen1p [12]. SETX gene mutations were identified in autosomal recessive AOA2 [5,13-17], autosomal dominant ALS4 [6] and more recently in autosomal dominant tremor/ataxia syndrome [18]. Missense mutations have been identified both in the C-terminus conserved helicase domain and the N-terminal part of the SETX protein, thus indicating that other than the helicase regions of the protein are also important for the pathogenesis of AOA2.

The homozygous c.5308-5311delGAGA mutation is a frame shift deletion that results in a Glu-to-Ile change at amino acid 1770 of the SETX gene and also introduces a stop codon 15 amino acids further down the new reading frame (p.Glu1770IlefsX15). It is likely that this mutation most probably results in a truncated protein lacking the c-terminal helicase domain of the SETX protein. This novel mutation adds to 30 already known SETX gene mutations causing AOA2, most of them reported by Fogel et al [16]. Identification of additional AOA2 mutations may contribute towards conclusive genotype/phenotype correlations.

We hereby describe the second ARCA mutation thus far identified in the Cypriot population after the FRDA GAA repeat expansion. Absence of this novel mutation from 12 Cypriot ARCA families and 37 sporadic cerebellar ataxia patients pending molecular diagnosis indicates that further genetic heterogeneity of cerebellar ataxia exists in the Cypriot population. The Cypriot population under study was 766,400 at the 2005 census; therefore, the frequency of patients with this novel SETX mutation is estimated to be 1 in approximately 150,000 of the population. Although the family excluded any known relation between the parents, a common ancestor or founder effect is more likely.

## Conclusion

We identified the second ARCA mutation in the Cypriot population, after the FRDA GAA repeat expansion, a novel SETX homozygous c.5308_5311delGAGA mutation that co-segregates with the ataxia phenotype in a Cypriot ARCA family with 5 affected individuals.

## Competing interests

The author(s) declare that they have no competing interests.

## Authors' contributions

PN, AG and CV carried out the molecular genetic studies and PN participated in drafting the manuscript. LTM and EZP were involved in ascertaining patients, providing phenotype data, obtained DNA samples and participated in editing the manuscript. KC conceived of the study, participated in its design and coordination, performed data analysis and drafted the manuscript. All authors read and approved the final manuscript.

## Pre-publication history

The pre-publication history for this paper can be accessed here:


